# Molecular Ecology in Health and Disease

**DOI:** 10.3402/mehd.v23i0.17302

**Published:** 2012-02-14

**Authors:** Tore Midtvedt

Why is *Microbial Ecology in Health and Disease* publishing a comprehensive 70-page review article on a simple molecule of interest to radiation protection? The article in question is: ‘Radiation protection following nuclear power accidents; a survey of putative mechanisms involved in the radioprotective actions of taurine during and after radiation exposure,’ by O. A. Christophersen (1).

My answer is as simple as the molecule itself: It is an excellent example of a molecule creating a completely new field of ecology that can be named molecular ecology in health and disease. It is one of the smallest amino acids, but it contains three atoms of oxygen and one of sulfur. It is produced by all mammals except cats, and it is used by human organisms for many reactions in many organs and in many types of cells.

In his review article, O. A. Christophersn has described the wide variety of these reactions as well as their physiological and patho-physiological consequences together with distinct therapeutic advice. His statements are anchored in 983 references, many of them going back to journals no longer easily accessible. Furthermore, the author discusses much more than the radioprotective action of taurine. In fact, he follows the molecule in and out of many types of human cells, even into the mitochondria. From an ecology point of view it is of particular interest that the molecule has an entero-hepatic circulation. Coming into the jejunum, the molecule may be attacked by microbial enzymes and may directly and indirectly impact on the intestinal microbiota.

Ever since I entered the field of intestinal microbiology close to half a century ago, I have been convinced of the crucial importance of the taurine molecule – in the intestinal tract as well as in the human body at large. I hope the present article may act as an eye-opener for many of our readers. Do not expect to understand all the effects this simple molecule may have, but try to lay bare its effects in your particular research field. Allow yourself to speculate a little – then hopefully you will recognize that you are entering the exciting and challenging field of molecular ecology in health and disease.

*Tore Midtvedt* Editor-in-chief
